# Combined Chylothorax and Chylous Ascites Complicating Liver Transplantation: A Report of a Case and Review of the Literature

**DOI:** 10.1155/2019/9089317

**Published:** 2019-07-21

**Authors:** Tommy Ivanics, Semeret Munie, Hassan Nasser, Shravan Leonard-Murali, Atsushi Yoshida, Shunji Nagai, Kelly Collins, Marwan Abouljoud, Michael Rizzari

**Affiliations:** ^1^Department of General Surgery, Henry Ford Hospital, Detroit, MI, USA; ^2^Department of Transplantation Surgery, Henry Ford Hospital, Detroit, MI, USA

## Abstract

Chyle leaks may occur as a result of surgical intervention. Chyloperitoneum, or chylous ascites after liver transplantation, is rare and the development of chylothorax after abdominal surgery is even more rare. With increasingly aggressive surgical resections, particularly in the retroperitoneum, the incidence of chyle leaks is expected to increase in the future. Here we present a unique case of a combined chylothorax and chyloperitoneum following liver transplantation successfully managed conservatively. Risk factors for chylous ascites include para-aortic manipulation, extensive retroperitoneal dissection, use of a Ligasure device, and early enteral feeding as well as early enteral feeding. The clinical presentation is typically insidious and may include painless abdominal distension. Diagnosis can be made by noting characteristic milky white drainage which on laboratory examination has a total fluid triglyceride level >110 mg/dl, an ascites/serum triglyceride ratio of >1 and a leukocyte count in fluid >1000/uL with a lymphocyte predominance. Chyle leaks may lead to significant morbidity and mortality. Numerous management options exist, with conservative nonoperative measurements leading to the most consistent and successful outcomes. This includes a step-up approach beginning with dietary modifications to a low-fat or medium chain triglyceride diet followed by nil per os with addition of total parenteral nutrition and somatostatin analogues such as octreotide. Rarely do patients require more invasive treatment. Early recognition and appropriate management are imperative to mitigate this complication.

## 1. Introduction

Chyle leaks, manifesting as chylothorax or chyloperitoneum/chylous ascites, can be broadly categorized into non-traumatic and traumatic etiologies. The latter can occur as a result of surgery. Chylous ascites is a rare complication after liver transplantation with reported incidences of 0.6-7% [1–7]. Chylothorax after abdominal surgery and liver transplantation in particular is even more rare [8, 9]. There appears to be a correlation between incidence of chyle leaks and the extent of surgical resection [10]. The incidence of this complication is likely to rise in the future with increasingly aggressive surgical resections, especially those involving the retroperitoneum, inferior vena cava reconstructions, lymph node dissections or in the setting of post neoadjuvant therapy [11]. While the majority of chyle leaks can successfully be managed conservatively, they can dramatically prolong length of hospital stay [10] and result in significant morbidity and mortality. Taking into account all these factors highlights the importance of early recognition and appropriate management of this rare complication.

We herein present a unique case of combined chylothorax and chyloperitoneum following liver transplantation successfully managed conservatively.

## 2. Case Description

The patient is a 56-year-old female with end-stage liver disease (true MELD score 12, exception MELD score 22) secondary to chronic hepatitis C genotype 1 likely contracted secondary to blood transfusion after a motor vehicle accident 40 years prior who presented for a deceased donor Liver transplantation. She had a 35-pack year smoking history. Preoperative morbidities included grade 2 esophageal varices, portal hypertensive gastropathy, encephalopathy and fluid overload as manifested by ascites and hydrothorax. She required regular therapeutic paracenteses every 3 weeks and weekly thoracenteses prior to her transplant.

The liver donor was positive for the hepatitis C virus and deemed high risk according to the Center for disease control and prevention (CDC) criteria. The cause of death was determined to be due to heroin overdose.

She underwent an orthotopic Piggy-back liver transplant with a duct-to-duct biliary reconstruction with stent and cholecystectomy. Additionally, an abdominal transdiaphragmatic thoracentesis was performed for drainage of a right-sided hydrothorax. Ultimately, a right-sided chest tube was placed due to significant persistent right-sided hepatic hydrothorax. Two 19F blake drains were placed in the perihepatic space. Cold ischemia time was 6h 44min. Warm time was 33 min. Total operative time was 7h 13min.

Her early postoperative course was unremarkable and she progressed appropriately. She was extubated shortly after surgery and her liver function tests (LFTs) trended down. Postoperative day (POD) 1 a liver ultrasound was obtained and demonstrated patent vasculature with normal directional flow with resistive indices of 0.72-0.77. The chest tube was placed to water seal on POD3 and subsequently removed along with the abdominal drains. She developed an acute kidney injury and a hypotonic, hypovolemic hyponatremia which was attributed to intravascular volume depletion which improved with sodium bicarbonate and fluid restriction. She was discharged home with home physical therapy on POD11. Her immunosuppressive therapy included thymoglobulin induction (POD1,3,5). Steroid taper began on POD1. Tacrolimus was initiated on POD3. She received perioperative antibiotic prophylaxis.

**Figure 1:**
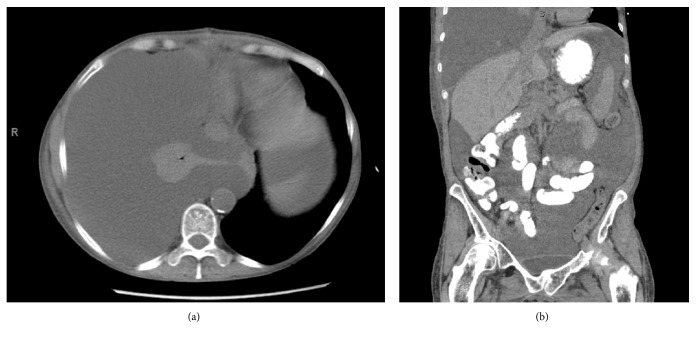
(a) Axial CT demonstrating large right pleural effusion with right lung atelectasis (b) Coronal CT scan demonstrating right chylothorax and chylous ascites.

**Figure 2:**
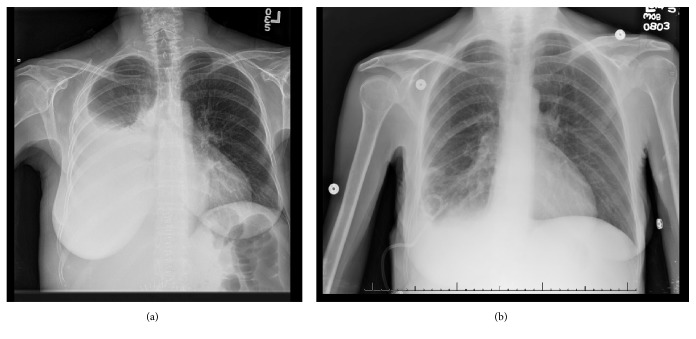
(a) Large right pleural effusion with significant right lung atelectasis and (b) subsequent Improvement of Right pleural effusion after pigtail placement.

**Figure 3:**
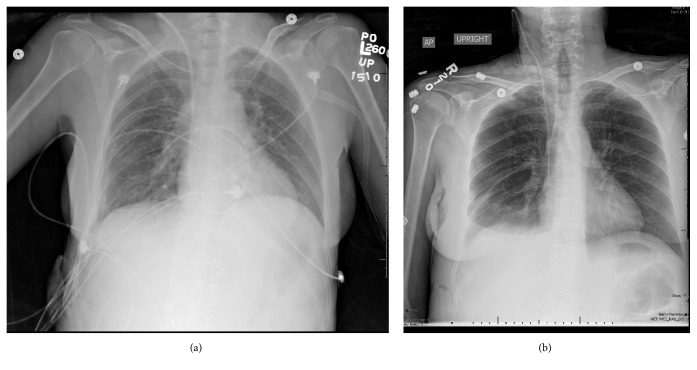
Chest x-rays with chest tube in situ and after removal of chest tube.

**Figure 4:**
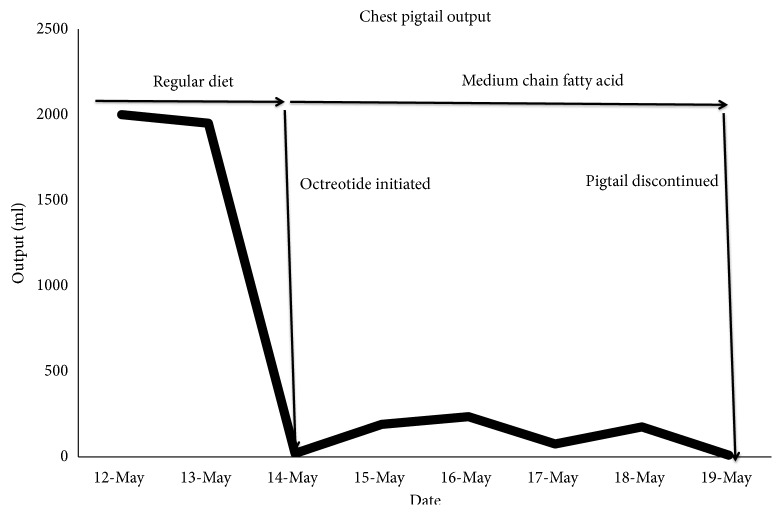
Chest pigtail catheter output over time.

Two days after discharge she was seen in clinic at which time a chest x-ray demonstrated presence of a large right hydrothorax/pleural effusion (Figure 1). She underwent a right-sided thoracentesis as an outpatient which yielded 1750 ml of cloudy yellow/amber fluid. Approximately one month thereafter she presented to the emergency department with abdominal pain, nausea, and constipation (Figure 1). Due to a re-accumulation of pleural fluid she underwent a right thoracentesis which now yielded 2000 ml of milky fluid. The pleural fluid analysis demonstrated the percentage of lymphocytes as 85%, triglyceride concentration was 1059 mg/dl, cholesterol <50 mg/dl, amylase 12 IU/L. There was no growth of any organisms. Similar proportions were found on the peritoneal fluid with a triglyceride level of 1043 mg/dl. These were deemed to be consistent with a chylothorax and chylous ascites, respectively. A pigtail catheter was placed in the right chest and an additional 1500 ml milky fluid was drained (Figure 2). A paracentesis was performed at the same time which yielded 2000 ml of similar milky fluid.). Additional paracenteses were performed and similar amounts of milky fluid were obtained. She was placed on a low fat diet and octreotide was initiated. The chylothorax improved and the pleural drain was removed within one week of presentation. (Figures 3 and 4). She has not required any additional interventions for her chylothorax or chylous ascites and continues to do well 4 years following her liver transplant.

## 3. Discussion

Chyle leaks can occur through various mechanisms, one of them due through direct leakage (such as lymphatic disruption after trauma or surgery) [11]. They can occur in various organ spaces such as the peritoneum (chyloperitoneum), pleural space (chylothorax), pericardium (chylopericardium), and mediastinum (chylomediastinum) [11, 12]. Chylous ascites after liver transplant may occur as a result of increased hepatic lymph production in the setting of portal hypertension and cirrhosis [11, 13] as well as inadequate perioperative sealing of lymphatic vessels [4]. In our case the combination of chylothorax and chyloperitoneum may have several explanations. Chylothorax has been described after thoracostomy tube placement but is exceedingly rare [14]. Chylothorax has been reported as a rare manifestation of cirrhosis as a result of transdiaphragmatic passage of chylous ascites which may even occur without appreciable ascites if the flow of ascites into to pleural space equals the rate of ascites production and be facilitated by pores in the diaphragm. [13, 15, 16] Intraoperatively, an iatrogenic transdiaphragmatic communication was created between the pleural and peritoneal space to alleviate the patients hydrothorax. This defect, if inadequately closed, may have led to direct translocation of chylous ascites into the pleural cavity. Assuming that the diaphragmatic defect was adequately closed, the more likely explanation for the chylothorax, however, is through the same mechanism as a hepatic hydrothorax occurs.

The clinical presentation of chyle leak is typically insidious and related to the anatomic location with painless abdominal distension and dyspnea as presenting symptoms in chylous ascites and chylothorax, respectively. The diagnosis is made by a combination of drainage of milky white fluid which may have a temporal relation with initiation of enteral feeds, total fluid triglyceride (TG) level >110 mg/dL, ascites/serum TG ratio of >1, leukocyte count in fluid >1000/uL with a lymphocyte predominance [4, 17]. Additional diagnostic modalities include computed tomography, lymphoscintigraphy, lymphangiography (considered the gold standard), laparoscopy, and laparotomy [11]. Lymphatic imaging can be used to identify the location of a leak. The consequences of chyle leaks can be major and lead to protein loss, immune compromise (lymphocytopenia), and electrolyte abnormalities such as hyponatremia and hypocalcemia [12, 18]. These may have detrimental consequences especially in a postoperative setting.

Risk factors for chylous ascites after Hepatobiliary surgery and liver transplantation include para-aortic manipulation, retroperitoneal invasion, early enteral feeding (defined as enteral feeds through jejunostomy tube within 24 hours of operation), presence of pretransplant ascites and use of LigaSure vessel sealing system (LVSS) for perihepatic dissection.[2, 3]. In our case a number of potential risk factors exist for the development of chyle leak exist. Firstly, liver transplant in and of itself may lead to chyle leak due to perihepatic dissection. Portal hypertension contributes to an elevated risk by increased production of hepatic lymph. Additionally, traumatic chest tube placement has been reported as a rare cause of chylothorax. Whether the chylous ascites leads to the development of chylothorax due to the preexisting peritoneopleural gradient which existed in this patient preoperatively or whether these two were separate occurrences is not clear.

The management options are numerous, with conservative enteral management resulting in the most consistent and successful outcomes with resolution of up to 100% of cases with multimodal therapy. This entails typically a step-up approach beginning with a low-fat or medium chain triglyceride (MCT) diet, as these are not absorbed through the lymphatics, and dietary cessation of long chain triglycerides (LCT) leads to a significant decrease in the rate of lymphatic flow promoting closure of any leak. The next step is making the patient nil per os (NPO) with the addition of total parenteral nutrition (TPN) followed by octreotide, a somatostatin analogue [1, 3, 4, 19]. The mechanism of Octreotide is thought to be through splanchnic vasoconstriction and decrease in hepatic venous flow with resultant decrease in gastrointestinal secretions and absorption and ultimately chyle flow [19]. Serial paracenteses are performed as needed for symptom relief [11]. Patients in whom conservative approaches have failed and continue to have high output leaks such as 1.5L/day or with rapid deterioration in nutritional status may be considered for more invasive therapies [12]. These include peritoneovenous shunts (such as Denver and LaVeen [20]), TIPS, interventional radiologic embolization, VATS with thoracic duct clipping/ligation or pleurodesis [11, 12, 18]. No randomized clinical trials exist comparing any of these treatment modalities which is likely due to the rarity and heterogeneity of the disease.

Chyle leak is a rare complication of surgical interventions which is expected to increase with expansion expanded indications for neoadjuvant therapy and retroperitoneal and transplant surgery. Additionally, increased use of diagnostic imaging may also contribute to rise in the rate of diagnosis of chyle leaks. Consequences of chyle leaks may be major and lead to significant prolongation in length of hospital stay, morbidity, and mortality especially with delays in recognition and management. There is significant overlap in the treatment of various anatomic locations of chyle leaks and prior surgery in one anatomic compartment does not preclude a chyle leak in another. The majority of patients see improvements with a step-up approach of conservative therapy and infrequently require invasive treatment.
